# Developing population health research priorities in Asian city state: Results from a multi-step participatory community engagement

**DOI:** 10.1371/journal.pone.0216303

**Published:** 2019-05-01

**Authors:** Julian Thumboo, Sungwon Yoon, Sharon Wee, Cheng Teng Yeam, Edwin C. T. Low, Chien Earn Lee

**Affiliations:** 1 Regional Health System, Singapore Health Services, Singapore, Singapore; 2 Department of Rheumatology and Immunology, Singapore General Hospital, Singapore, Singapore; 3 Programme in Health Services and Systems Research, Duke-NUS Medical School, Singapore, Singapore; 4 Duke-NUS Medical School, Singapore, Singapore; 5 Changi General Hospital, Singapore, Singapore; Aga Khan University, KENYA

## Abstract

**Objectives:**

To identify a broad range of research priorities to inform the studies seeking to improve population health outcomes based on the engagement of diverse stakeholders.

**Methods:**

A multi-step, participatory and mixed-methods approach was adopted to solicit and structure the investigative themes from diverse stakeholders. The priority setting exercise involved four key phases: (1) feedback from community leadership; (2) interim ranking survey and focus group discussions during the population health symposium; (3) individual in-depth interviews with stakeholders in the community; and (4) synthesis of the research priorities from the multistep process.

**Results:**

Diverse stakeholders in Singapore, comprising community partnership leaders, health care and social service providers, users of population health services, patients and caregivers, participated in the research priority setting exercise. Initial 14 priorities were identified from six community leadership feedback, 42 survey responses, two focus groups (n = 16) and 95 in-depth interviews. The final integrated research agenda identified six priorities: empower residents and patients to take charge of their health; improve care transition and management through relationship building and communication; enhance health-social care interface; improve respite care services for long-term caregivers; develop primary care as a driving force for care integration; and capacity building for service providers. Selected research questions in each priority area were also generated to develop novel models of care, foster collaboration, implement optimal services and enhance understanding of the end users’ care needs.

**Conclusions:**

This study illuminates that greater community engagement in research priority setting for population health can facilitate the formulation of evidence-based research agendas that matter to the care providers and service users in the community. The outcomes derived from this exercise will help focus researchers’ efforts through which meaningful gains can be made for population health.

## Introduction

There is a growing recognition of the importance of community involvement in setting the strategic directions of population health research agendas. In the past, the role of the community and public (e.g. patients, residents, care providers) has been restricted to being end users and beneficiaries of health and social research whereas decision-making on what research is conducted has been the domain of a small group of experts, mainly researchers. This clear task division seems evident not only in clinical research in general but it can be also observed in population health research. In the past decade, however, active involvement of the public and patients in health research has been increasingly advocated by major research funding programmes in western nations [1–3].

A growing body of research indicates that engagement of end users in health research is found to promote positive health outcomes and alleviate health inequity [4–8]. Through the formation of partnerships and reciprocal learning, inputs from members of public and patients can increase insider perspectives, thereby guiding the development of interventions that is well aligned with the needs of healthcare users. Working collaboratively with patients in clinical research can also lead to better understanding of healthcare services and treatments pertaining to particular health conditions [9, 10].

Various methods have been employed to involve users in health research including citizen juries, questionnaires, focus groups, rapid appraisal techniques, neighbourhood committees, community forums and consensus conferences all aiming to identify perspectives, needs and priorities of community [11–14]. Models of engagement have been conceptualised as incorporating different levels, on a ladder or continuum, ranging from the provision of information to research users through consultation, co-production and delegated power to full user-control [15–16]. Studies also showed that research user engagement occurs in various stages of research process from research programming and design to research evaluation and dissemination of the research results [17–18].

One of the important stages of the research process for the members of the public to be involved in is that of research priority setting. For population health, in particular, public involvement can help effectively target research programmes that will provide the greatest benefit to public health and maximises the impact of investment for resources. Despite the widespread use of public involvement as an element of research practice, the majority of studies have predominantly focused on engaging specific population subgroups or patients with chronic diseases (e.g. older people, young people, people with cancer, COPD, asthma, spinal cord injuries, stroke, mental health problems) in different stages of research process (e.g. research design, data collection, interpretation, secondary research) [19–24]. There have been relatively few published literature on eliciting views of the public and community to inform the strategic direction for general health research agenda. For example, priority setting exercises conducted in US and UK appeared to have focused on broad health agendas such as social determinants of health and service delivery [25–26].

In light of the dearth of literature on public involvement in the direction of population health research, we initiated a research priority setting exercise by engaging community members. Community members are defined in this exercise as users of population health services, health and social care providers, community leaders, advocates, affected communities and voluntary welfare organisations [27–28]. In Singapore where this study was conducted, the government promotes a ‘many helping hands approach’, a framework based on mutual help, reciprocity and social capital in the response to rapidly ageing population and economic volatility [29]. Under this approach, various community health and social services are offered to empower individuals and families in need and build a strong community. These include providing care for the older people and disabled, counseling for individuals and families in need, support for the destitute and low-income families, rehabilitation and nursing patients in their homes, community mental health services, end-of-life care and primary care services among others. At present, more than 10,200 community care professionals are involved in quality care delivery playing a pivotal role in supporting the health of population [30]. Prior to the initiation of priority setting exercise, there existed little collaborative efforts to foster knowledge exchange and transfer [29–31] between researchers and community members, the user of research evidence, in the prioritization of studies seeking to improve population health outcomes.

The overall aim of this exercise was to establish a broad range of research priorities to inform the studies seeking to improve population health outcomes through an integrated, multi-step, participatory and mixed-methods approach. Greater community engagement in research priority setting can not only facilitate the formulation of evidence-based research agendas, but this process would also empower community partners and members of the public to identify opportunities and strategies for change by building on their knowledge, input and lived experience. This paper presents the key findings from the work.

## Methods

Ethics approval was obtained from the SingHealth Centralised Institution Review Board, Singapore. Written informed consent was obtained from participants.

### Setting

Singapore is a developed Asian city state in South-East Asia with a multi-ethnic population of 5.6 million people. Singapore’s healthcare financing system employs a mixed model of public and private funding to ensure the right balance between individual responsibility and social protection. In 2018, the Singapore government put aside S$10.2 billion for healthcare expenditure [32]. While Singapore’s annual healthcare spending (4% of the Gross Domestic Product) is much lower than its counterpart in many developed economies (16% in the USA, 8% in the UK), health outcomes of Singapore are largely comparable with these countries. Singapore’s population is rapidly ageing. The proportion of residents aged 65 years and above has increased from 7% in 2008 to 13.7% in 2018. By 2030, this figure is expected to be doubled to 27%. Healthcare expenditure is projected to increase from S$10 billion in 2016 to S$12 billion in 2020 [33].

### Process of data collection and analysis

**Fig 1:**
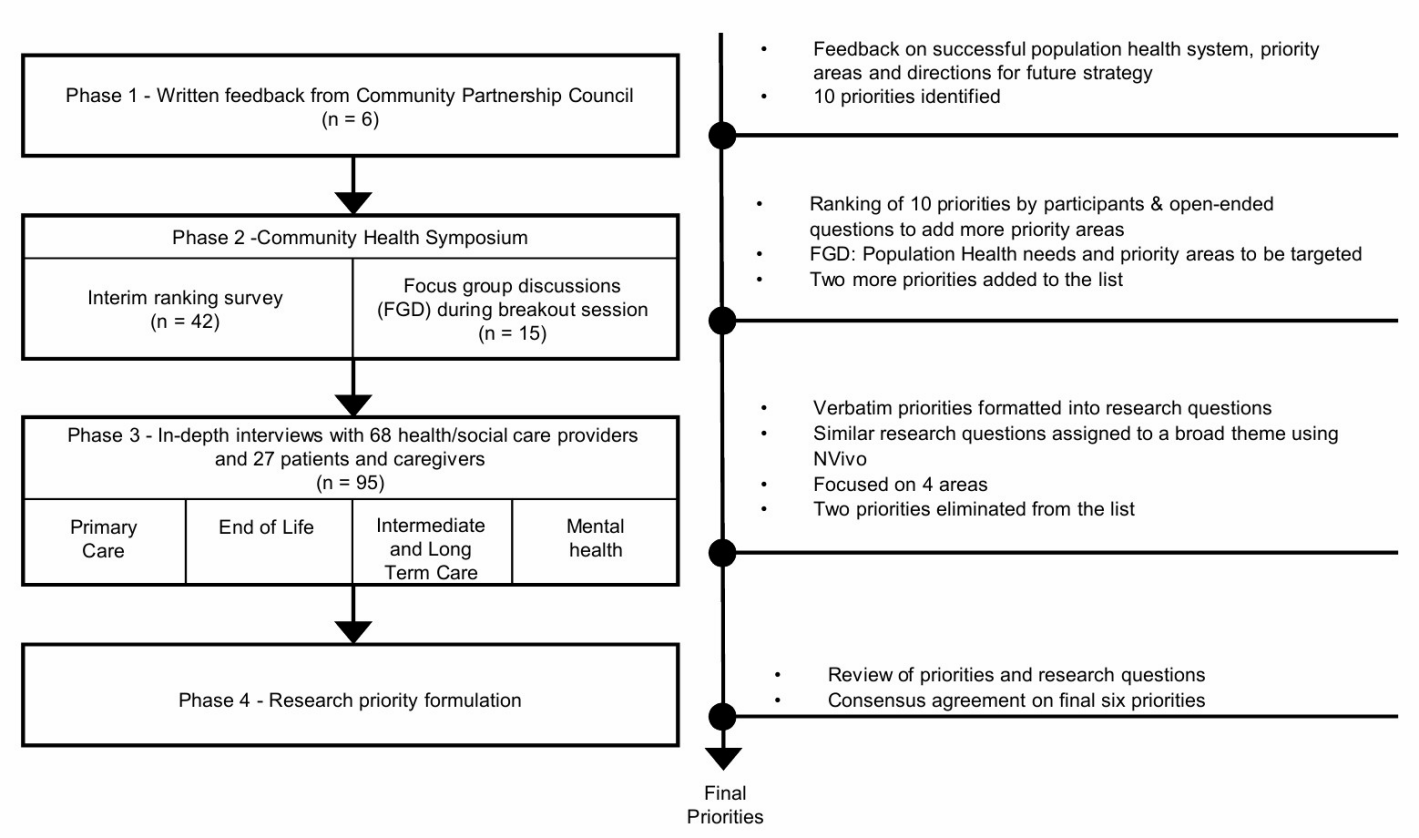
Summary of methodology.

The study adopted a multi-step process to solicit and structure the investigative themes from diverse stakeholders (summarised in Fig 1). The stakeholders in this study included health and social care providers, representatives from the SingHealth Regional Health System (a public health care entity overseeing health of one third of the Singapore population), government agencies, voluntary welfare organisations and private sectors and users of population health services (i.e. patients, caregivers and volunteers). The multi-step process included feedback from community leadership, an interim ranking survey and focus group discussion during the population health symposium and individual interviews with stakeholders in the community. Engaging community for setting population health agendas was a new enterprise in Singapore. This multistep process incorporated a two-pronged approach to participant recruitment: 1) tapping on existing channels such as formal meetings and events pertaining to population health; 2) referrals from known contacts working in population health. Phase 1 and 2 employed the first approach while phase 3 employed a combination of the two approaches. At each data collection point, participants were informed about the importance of shared understanding and the need for engaging community members in research priority setting to improve the usefulness of research and population health outcomes.

#### Phase 1—Community leadership feedback

The first stage of the study involved collation of feedback from members of the Community Partnership Council (CPC), a core leadership group of community partners, consisting of 16 individuals (e.g. directors and heads of health and social care organisations; foundations; national councils; government agencies). The CPC members were approached via email inviting them to provide feedback on what constitutes a successful population health system, priority areas for improvement and directions for future strategies. A second reminder was sent one week after the initial request. To develop a composite list of research priority areas across CPC members’ feedback, a process for grouping similar clusters of topics was undertaken using a form of thematic analysis based on grounded theory approach [34, 35]. This process involved independent assessment of all written feedback by two researchers followed by repeated discussion for each category among research team members producing a list of provisional priority areas.

#### Phase 2 –Interim ranking survey and focus group with stakeholders

In the second stage, we carried out an interim ranking survey, comprising 10 broad categories of priority area, distilled from CPC feedback, to generate a more concise list. The survey questionnaire was disseminated during a community partners’ symposium on population health held in January 2018, which approximately 120 community care providers as well as patients and caregivers attended. Participants were asked to rate each of the priority areas using a 5-point Likert scale of importance (i.e. range from ‘very important’ to ‘not important’). Additionally, we provided an open-ended question in which participants were asked to add, if they wished, priority areas not listed in the questionnaire and provide further text justifying the importance of the area. The questionnaire responses were analysed using descriptive statistics (e.g. frequencies, means). For open-ended questions, we carried out a thematic analysis of written comments by reviewing them for emerging themes and categorising the comments according to the main themes.

In parallel with the interim prioritization survey, two focus group discussions were conducted during the breakout session of the symposium. The group discussions were aimed at exploring the population health needs and challenges and eliciting participants’ views of priority areas to be targeted for improving population health. Due to the logistic issues, the focus group sessions were not audio-recorded. However, in order to inform subsequent lines of enquiry, notes were taken by two scribers and flip-charts of the main points contributed by participants were retained.

#### Phase 3—In-depth interviews with key informants

The third stage of the priority setting exercise was in-depth interviews with community members in a single face-to-face meeting to further explore emergent concepts about population health priority and identify divergent perspectives. Participants were purposively identified through a mixture of the following methods: from the list of community partnership council and survey respondents; through known contacts working in the area of population health; and a respondent-driven snowball sampling technique. Following the identification of participants, we sent an email invitation. A maximum variation sampling strategy was used to ensure that participants represented a range of stakeholder groups and organisations within Singapore. They included community partners from voluntary welfare organisations, healthcare professionals (clinicians, nurses, and allied health professionals), social service providers, interested patients and caregivers and other service users in the field of population health.

A trained interviewer in qualitative research conducted interviews with those who agreed to participate between February and November 2018. Interviews took place at a location of the respondent’s choice, which for the majority of participants was at their place of employment or home. Consent forms were completed on the day of the interview. We developed an interview guide with open-ended questions to solicit participants’ views of gaps in their areas of work (care providers) or services (service users) and suggestions for improvement. We also asked them to reflect on the priority areas and rankings collated from previous phases and suggest new priorities, if any. Each participant was also asked to identify and frame a more specific research question addressing a specific priority area of their choice. For some participants, it was onerous and inhibiting to be asked to formulate research questions in such a way that they could be tested in a study. In that case, we asked for suggestions for research topics only in more general terms. Each interview lasted approximately 30–90 minutes.

All interviews were audio recorded and transcribed verbatim. We achieved data saturation after 12 interviews in each area of population health (primary care; mental health; end of life care; intermediate & long term care). The interview data were analysed using constant comparative method for the thematic analysis. Two coders independently read and coded the transcripts. Coded segments were then re-analysed, coded into subcategories, and compared again. All authors had consecutive rounds of iterative discussion, through which any discrepancies in interpretation were reconsidered in order to reach an agreement. This allowed for inter-coder clarification of research themes and research questions within these themes, thus enhancing validity and reliability. NVivo 11 (QSR International), a software package for qualitative data analysis, was used for data management and coding. For rigour and transparency, we anchored our methodology according to the Consolidated Criteria for Reporting Qualitative Research (COREQ) checklist [36] (S1 Table).

#### Phase 4—Research priority formation

The final analysis involved synthesising the research priorities from the multistep process that included drawing upon thematic analysis from the CPC feedback, integrating these themes with the information from survey data and focus groups and examining thematic analysis of in-depth interviews and associations between key informant types and research priorities. Using a triangulation model, all collected data were analysed independently by two coders and discussed among the research team to achieve concurrent triangulation. Specifically, the potential questions raised by participants were first put into categories by theme. Where a question could belong in more than one theme, a judgement was made as to the respondent’s primary intended questions. At the end of the process, a list of research questions was agreed upon based on two criteria: a) members of the research team agreed that final research themes/questions were a true reflection the question intended by the respondents; b) the question had been proposed in some form by at least 10 respondents across different phases.

The study was approved by the SingHealth Institutional Review Board.

## Results

### Participant characteristics

For feedback from CPC (i.e. phase 1), we approached 16 individuals and six responded to our request, providing their comments on population health priorities. For phase 2, 42 out of 126 individuals attending the community partners’ symposium participated in the interim ranking survey. Half of the participants were health care professionals working in the community. Participants had on average 9 years of experience in the field of community/population health (Table 1). The participants of the first focus group (n = 8) consisted of healthcare professionals, researchers, a government official and community partners in areas of chronic disease management, care of older people and end of life care while the second group (n = 12) included healthcare professionals, Regional Health System (public health care entity) staff, nurses involved in care management and managers of voluntary welfare organisations. Of 42 participants in the ranking survey, three took part in the focus group discussions (i.e. two healthcare professionals and one community partner). For phase 3, a total of 95 individuals consisting of 68 care providers and 27 service users participated in the in-depth interviews. We approached 75 care providers and 68 agreed to take part in our interview request. Reasons for refusal included being unavailable and non-response to our invitation. Approximately 68% were female. More than half of care providers were healthcare professionals (57%) while 46% of them were in the leadership role of their organisation. As for service users, 36 individuals were approached primarily through referrals from care providers and 27 individuals agreed to participate in the interviews. Major reasons for decline were disinterest and busy schedule. Approximately 65% were female and more than two-thirds of them were Chinese (Table 2).

**Table 1:** Phase 2—Participant characteristics of interim ranking survey (n = 42).

Role	N	(%)	Years of care experience (mean)
Doctor/ Nurse/ Allied Health Professional	21	50.00	10.26
Social service provider	9	21.43	5.69
Regional Health System staff	9	21.43	4.61
Patient/ Caregiver/ Volunteer	3	7.14	16.83

**Table 2:** Phase 3—characteristics of service users and service providers in in-depth interviews (n = 95).

	Service Users (n = 27)		Service Providers (= 68)	
**Gender (%)**				
Male	9	(33.33)	22	(32.35)
Female	18	(66.67)	46	(67.65)
**Area of work (%)**				
Primary care	12	(44.44)	14	(20.59)
Mental health	3	(11.11)	19	(27.94)
End of life care	6	(22.22)	14	(20.59)
Intermediate and long-term care	6	(22.22)	21	(30.88)
**Ethnicity (%)**				
Chinese	18	(66.67)	-	
Malay	7	(25.93)	-	
Indian	2	(7.41)	-	
**Highest Level of Education (%)**				
No formal qualification	2	(7.41)	-	
Primary	3	(11.11)	-	
Secondary	3	(11.11)	-	
Tertiary or above	19	(70.37)	-	
**Profession (%)**				
Healthcare professional	-		39	(57.35)
Social Service Provider	-		29	(42.65)
**Institution (%)**				
Government	-		1	(1.47)
Public	-		33	(48.53)
Private	-		5	(7.35)
Voluntary Welfare Organisation	-		29	(42.65)
**Years of experience (%)**				
Less than 5 years	-		10	(14.71)
5 to 10 years	-		23	(33.82)
11 to 15 years	-		7	(10.29)
16 years or more	-		28	(41.18)
**Type of role (%)**				
Leadership	-		31	(45.59)
Operational	-		37	(54.41)

### Views of leadership on population health priorities

The CPC feedback yielded a broad range of priority areas. Respondents indicated that a successful population health system should empower residents to take charge of their health so that they, with support from community services, can live healthy and meaningful lives. Many stated that a person-centric approach, collaborative partnerships through a network of partners and effective communication by sharing standardized protocols were critical to success for population health. Other priority issues included strengthening primary care, increasing community outreach to end-of-life issues as well as supporting caregivers of long-term chronic patients. In order to facilitate the next stages of the project, the research team summarised the comments thematically and sorted them into ten priority categories. They included: empowering residents; establishing a network of collaborators; improving effective handover; enhancing information sharing and communication; improving respite care services for caregivers; developing primary care; harnessing existing resources; increasing outreach to end-of-life issues; enhancing work experience of care providers; and developing a use case.

### Interim ranking survey and focus group discussions from community partners

Table 3 presents the participants’ ratings of their views about the ten key priority areas, generated from comments of the CPC. In general, apart from the item on “developing a use case” (M = 3.40±1.71), the remaining items had mean scores of 4 and over indicating that the vast majority of key priority areas were perceived important by survey respondents. The highest rated priority areas were “empower residents to take charge of their health” and “establish a network of collaborators/community partners with defined roles.” The lowest rated priority areas were “enhance the work experience of social and health care providers” and “develop a use case by engaging health and social care partners.”

**Table 3:** Findings of the phase 2 –ranking exercise and additional priorities.

Rank	Priority area	Mean (SD)
1	Empower residents to take charge of their health	4.79 (0.56)
2	Establish a network of collaborators and partners with defined roles	4.62 (0.79)
3	Improve effective handover between partners, and localised plan for individuals	4.52 (0.92)
4	Enhance information-sharing and communication across health and social care sectors	4.43 (1.25)
5	Improve respite care services for mental wellness of long-term caregivers	4.36 (1.03)
6	Develop primary care as a driving force behind the affordable health care	4.31 (1.20)
7	Harness existing community resources such as building peer supporters and volunteers	4.24 (1.23)
8	Increase outreach to social and health community on end-of-life issues and dying well	4.14 (1.20)
9	Enhance the work experience of social and health care workers	4.10 (1.34)
10	Develop a use case by engaging health and social care partners	3.40 (1.71)
Newly added item	Provide a personalised care that enables residents to stay well and achieve the best health outcomes	
Newly added item	Segment population into mutually distinct groups for targeted interventions	

Participant feedback in open-ended questions highlighted additional research themes, which were added to the 10 research priority areas from the phase 1 community leadership feedback: one was to *provide a personalised care that enable residents to stay well and achieve the best possible outcome*. Focus group discussions conducted during the breakout session similarly reflected on the need for adopting tailored and holistic care by incorporating patients’ social and health care needs. The focus group discussions also yielded a new priority—*need for segmenting the population into mutually distinct groups for targeted interventions*—which was added to the research priority areas.

### Findings from in-depth interviews with key informants

In-depth interviews with key informants generated additional two priority areas (*enhance community resources and capacity to prevent hospitalisation and institutionalisation*; *increase public and provider awareness of illness with stigma*). By and large, there was considerable similarity in the priorities between the results of the phases 1 and 2 and those of the in-depth interviews. Therefore, the in-depth interviews did not significantly alter the research priorities identified in the previous phases. However, some items identified in previous phases did receive very little or no input from participants, thereby being grouped as ‘no input’ items (*Harness existing community resources such as building peer supporters and volunteers; Develop a use case by engaging health and social care partners*). The wording of the priorities was also reviewed with participants and in some cases, revisions were suggested for clarity. Altogether, twelve themes were identified for inclusion in the phase 3 priority setting exercise. Table 4 illustrates each of these themes with reference to quotes from in-depth interview transcripts.

**Table 4:** Findings of the phase 3 –key priorities and illustrative quotes.

Priority	Illustrative quotes
Empower residents to take charge of their health	“I think why we have a problem on diabetes, the “war on diabetes”, is that there is a lack of understanding of what diabetes can do to a person beyond just oral medications. Even in our direct encounters with patients, they have very poor lifestyle habits.” (service provider #32, primary care) “For certain medications, patients can titrate themselves, they can stop taking if they have this symptom for example. So rather than telling patients to come back to hospital if they have problems…more education is needed…we need to build the rapport.” (service provider, # 20, intermediate & long term care)
Establish a network of collaborators/community partners with defined roles	“I think we [care professionals] should be aware of each other. Maybe we should come out with a list of community partners so that we know which organisation would be responsible for certain things.” (service provider #2, intermediate & long term care) “I find networking very important for us. You can meet multiple partners and share knowledge and experiences. Networking is a platform to communicate.” (service provider #65, end of life care)
Improve effective handover between partners, and localised plan for individuals	“If our client [elderly patient] is discharged, it would be great if the hospital can inform us [senior activity centre]. Let’s say his living condition is so bad. The hygiene of his house is not there. He is on wheelchair. His meals are not in place…what will happen to him if he is discharged?” (service provider #5, intermediate & long term care) “I feel that it is not so much about what we want to give to the elderly, but about what they need. Sometimes they have different opinions and we tend to push for ours.” (service provider #35, intermediate & long term care)
Enhance information-sharing and communication across health and social care sectors	“The next thing we [VWO] should be doing is to work with the other VWOs. I don’t know how to start…maybe we can organise a regular platform to share information such as workshops.” (service provider #47, intermediate & long term care)
Improve respite care services for mental wellness of long-term caregivers	“I think for the younger generation, they have so-called camps or holiday programmes. Caregivers should have a time off from home on weekend. It is already a respite for them.” (service provider #19, mental health) “I think this [respite services] is lacking in our system right now. Respite care not just for mental illness but also for more general illness…we need night respite, day respite etc.” (service provider #7, end of life care)
Develop primary care as a driving force behind the affordable health care	“The information, the medical record the private GP has is not linked to the hospitals. You got to bring your records over to the hospital. If you lose your medical records, then they [hospital] will ask you to do another round of x-rays and tests.” (service user # 12, primary care) “As a primary care liaison, I wish we can do better in terms of being fully aware of our community partners. How do we draw in primary care as part of the team as we are talking about population and stratification?” (service provider #51, primary care)
Increase outreach to social and health community on end-of-life issues and dying well	“Had they [acute hospital] told us earlier about the duration of [my wife’s] life span and all the information, we would have checked out various options and gotten things in place much earlier. At the almost last stage, I didn’t even know what palliative care was.” (service user #3, end of life care) “It [end of life issue] is more of social norms kind of situation…there are other social implications because people have this notion that I should leave my dying in the hands of my children, because this is the filial thing.” (service provider #17, end of life care)
Enhance the work experience of social and health care providers	“We need to improve the competency of community mental health providers for a proper psychological intervention and assessment. This is a missing gap we noticed in most of our VWOs.” (service provider #31, mental health) “I hope government will change policies to retain trained foreign nurses because we really need them in our nursing home.” (service provider #40, intermediate & long term care)
Provide a personalised care that enables residents to stay well and achieve the best health outcomes	“Our model of care is a patient-centred care approach. So that comes under the priority of empowerment of patient and caregivers.” (service provider #27, end of life care) “Everybody is unique in their own ways. We should view their values in their perspectives….this is called empowerment and choice…availability of choices.” (service provider, #3, intermediate & long term care)
Segment population into mutually distinct groups for targeted interventions	“This is important but may be similar to the other priorities [empowerment of patients, personalised care]…” (service provider #29, end of life care) “I can prioritise a bit more for you. The empowerment of patients is linked with personalised care and targeted intervention.” (service provider # 61, mental health)
Enhance community resources and capacity to prevent hospitalisation and institutionalisation*	“We should do this community support aspect well, pump in more resources on that, so that people don't have to go back to hospitals.” (service provider #22, mental health) “When you run an institution, a residential set up is more costly than to provide community support. When patients are in the community, they can be active citizens of the society to contribute. Being the community will give them a sense of self-worth and meaning for life.” (service provider #55, primary care)
Increase public and provider awareness of illness with stigma*	“I have a client who got better but later on her condition deteriorated. For her, coming to our setting [mental hospital] again is like ‘oh no, it’s like I am facing back’…shame and guilt…I mean to address this issue, it is all about public awareness…de-stigmatise mental illness and set up more mental wellbeing clinics in the community that are readily accessible.” (service provider #59, mental health) “I think end of life care is a bit taboo. People are not comfortable talking about it when someone has cancer because they feel it destroys hope, make people feel depressed or give up. But if they’re not aware of the options available, they don’t know the goal of the treatment that is offered.” (service provider #45, end of life care)
Harness existing community resources such as building peer supporters and volunteers	No input from participants in Phase 3
Develop a use case by engaging health and social care partners	No input from participants in Phase 3

* Newly added item.

### Final priority list

From the results of the three phases of priority, the themes and suggested research questions were sorted and collated, which were then categorised into subthemes. The sub-themes were merged into six broad themes. The sorting of the responses resulted in considerable data reduction with similar questions being pooled together. The final list of the top six priority areas is presented in Table 5.

**Table 5:** Findings of phase 4—research priorities, themes and potential research questions.

Research priorities	Rationale for the choice by participants	Themes	Potential research questions to be answered
Theme 1: Empower residents/patients to take charge of their health	• Need for moving towards new models of care with patient-centred approach • Patient perspectives and needs overlooked • Empowerment improves chronic disease management	• Health education for members of community • Care needs of residents/patients • Avoiding medicalisation of social problems	• What behavioural modification interventions are effective for different segments of patients/residents? • How can we increase public awareness of personal responsibility for health? How effective are the existing education programmes on self-management? • How can we empower patients with usable tools, personal coaching and virtual care? • What are patients’ expectations and concerns? How to dispel misconceptions and misinformation? • What are the patient’s self-management goals and how do we support them?
Theme 2: Improve care transition and management through relationship building and communication	• Concerns about duplication and fragmentation of services • Limited understanding of one another’s missions and agendas across community partners • Reported challenges regarding the use of the national information system and competing IT platforms	• Sharing of patients’ information across care continuum • Communication involving all parties	• How can the various community partners work together more effectively? • Is there a role for common electronic platforms/apps to create an interactive network for community partners and related parties? Could these devices be used support patients and clients? • What are the barriers to utilising existing electronic information system such as NEHR (National Electronic Health Record) across the care pathways?
Theme 3: Enhance health-social care interface	• Disconnect in services between health and social care • Lack of information sharing due to absence of shared system	• Integrated health care and social services platform • Impact of social aspects of life and related support issues on health outcomes	• What is the core and targeted information needed by care providers involved in community health and social care? • How can we evaluate the performance towards the integrated health and social care system? (structure, function and outcomes and benefits for those who use services)
Theme 4: Improve respite care services for long-term caregivers	• Perceived shortage of available respite services • Lack of awareness of respite services • Need for support for caregiver wellbeing	• Caregiver training and resilience • Public awareness of illness with potential stigma (e.g. dementia, end of life) • Impact on family and others (caregiver depression and fatigue)	• How can we improve the resilience of long-term caregivers? What training is needed? • What are some available resources in the community to help manage the long-term caregivers? How can we effectively disseminate the information on community resources? • What is the effectiveness and cost-effectiveness of respite care programmes in supporting informal caregivers? • How do we facilitate the capacity of volunteers?
Theme 5: Develop primary care as a driving force for care integration	• Importance of primary care for population health • High patient load in public primary care • Limited involvement of private GPs in population health • Disconnect between primary care and community partners	• Care coordination • Continuity of care • Management of patients with complex care needs in primary care	• How do we evaluate factors that influence general practitioner’s decision to (or not to) refer patients to specialist care and community care? • What is the prevalence of primary care patients lost to follow-up in the system? What are the risk factors associated with loss to follow-up? • How can we streamline the prevention efforts in primary care? (e.g. diabetic and eye screening within one centre) • What are the challenges primary care providers face in managing complex patients in the community? What resources are required?
Theme 6: Capacity building for service providers	• Community care providers as central to population health • Perceived gaps in skill sets, knowledge and capabilities amongst community partners • Pre-eminence and appeal of specialist care & tertiary healthcare institution	• Awareness of community care services among specialists in acute hospitals • Upskilling of community care providers through training and education	• What are the perception, knowledge and awareness of community health and social care resources and mechanism among specialists in restructured hospitals? • Does training and upskilling of community care providers (e.g. nursing home health workers, community mental health workers) lead to improved care outcomes?

## Discussion

To the authors’ knowledge, this is the first paper to report the results of a systematically conducted multi-stakeholder priority setting exercise for research into general population health. By engaging community leaders, health care and social service providers, service users and other stakeholders together, this exercise demonstrated the importance of developing research agendas that are valued by community members. We employed a comprehensive, rigorous and inclusive process to define research agendas that matter to the community. Our findings were consistent with previous priority exercises that indicate that individuals with lived experience are capable of research prioritisation and that stakeholder involvement can help foster sharing of practical knowledge among stakeholders and broaden research agendas [2, 4–8, 19–24].

The results of our priority setting exercise support Singapore government’s *Healthy Living Master Plan* [37]. A key theme of the Plan was greater “engagement of the public” to foster a healthy living that is accessible, affordable and participatory. Our exercise clearly fulfils the theme. In particular, findings from in-depth interviews revealed the rationale for demedicalising social problems (e.g. social deprivation, isolation, financial insecurity) and prioritising research on the effective integration of health and social care services (theme 3 priority). It was generally felt that many research themes are best addressed by the coordinating activities between and among health and social care providers across care continuum. A common question raised was how to drive optimal models of care by fostering the process of community stakeholder engagement. It is expected that future studies underpinning the theme 3 priority may facilitate cross-sector research collaborations resulting in more research networks and interdisciplinary studies.

In Singapore, one in four adults aged 40 and above has at least one of the following chronic diseases–diabetes, high blood pressure, high blood cholesterol and/or stroke [38]. About one in three deaths in Singapore is also due to these chronic diseases [39]. Singapore has also one of the most rapidly ageing populations in the world, with 20% of residents becoming aged 65 and above by year 2030 [40]. Given that the prevalence of chronic diseases increases with age, this poses a huge challenge for future healthcare resources, an issue that is reflected strongly in our top list of research priorities. Empowering residents to take charge of their health through education and other means (theme 1 priority), coupled with improving patients’ care transition and management (theme 2 priority) not only might improve the health outcomes of long-term illness, but it would ultimately lead to sustained benefits while reducing the health care utilization and costs. Recent developments and research into virtual care, automation and wearables for chronic disease management are promising [41–43], and further research on novel models of care is clearly warranted.

Related to this, for patients with chronic mental illness, disability and rehabilitation, care is often delivered not through health care institutions but in the home by family members supported by community-based services. Indeed, the demand for home-based care is increasingly emphasised as the population ages [40], yet the supply of informal care is shrinking due to the rapidly changing family structure as well as significant emotional and physical burdens experienced by caregivers. Certain priorities, therefore, focused on the need to improve services that alleviate caregiver burden (theme 4 priority). Locally, a few studies have explored the burden of the family caregivers of patients with long-term illness [44–46], but the availability of respite care programmes and its impact on caregivers’ resilience remain relatively unknown. The design and implementation of a dedicated respite care service must consider the caregivers’ needs and preferences and its success could be assessed using a multi-faceted approach.

Some priorities were oriented towards care professionals. One particular area that received consistent suggestions was capacity building for care professionals in the provision of appropriate services (theme 6 priority). In-depth interviews with care professionals revealed that capacity building is not merely skills training, but it would also include the need to increase the awareness of community care services among specialist health care providers in acute hospitals to streamline and optimise care processes. On the other end of the spectrum, on-going training and continuing education, combined with a sustained channel for communication with specialist doctors, was the salient theme that emerged among community care providers. To develop optimal care for patients in the community, future studies should consider investigating the efficacy of improved awareness of community care services amongst specialists and/or upskilling of community care providers using both clinical and patient-centred outcomes.

A significant area of concern was the role of primary care and the general practitioners (GP) in population health. While Singapore’s public primary care system plays a gatekeeping role alongside partners from the social and community care sectors, approximately 80% of primary care is provided by a private sector comprising 2,400 general practitioner clinics [47]. The share of chronic disease management is however disproportionately distributed between the public and private primary care providers. It was reported that about 55% of chronic patients are managed by private medical clinics while the government-run subsidised polyclinics manage the remaining 45% of chronic diseases [48, 49]. This suggests that the costs of services could be a factor for patients choosing subsidized services since chronic disease management requires follow-up consultations and incurs higher medication and ancillary costs than acute primary care conditions. This issue is echoed in the priority list (theme 5 priority)—the need to strengthen the primary care systems especially when long-term management is required for chronic diseases. Therefore, increased emphasis should be placed on the assessment of current state of primary care systems in terms of access, care continuity and affordability, and how they can be improved. A recent development such as primary care network, a partnership amongst private GP clinics supported by a mobile government-funded team of nursing and allied health professionals, that aims to improve chronic disease models of private care is encouraging [50, 51]. Research community could consider a systematic approach to assessing how the new models of care are expected to slow down disease progression, reduce complication rate and in turn, minimize referrals to acute hospitals.

Innovative partnerships between healthcare stakeholders and researchers are steadily increasing providing an instructive illustration of collaborative approaches to improving population health [52]. This study adds to this growing trend in population health of fostering research priority setting partnerships to achieve better health outcomes. Our findings were in line with few published empirical studies on research priority setting that intersectoral coordination through systemic approach was a key element for success in population health [25, 26]. Therefore, regardless of healthcare settings, sustained efforts should be deployed to ensure that stakeholders across sectors share aligned visions and values to achieve the common goal for population health.

In evaluating the overall process of the community engagement in research priority setting, we learned that there is a considerable need to increase research literacy of the community so that community members can understand what is being asked for, and fully participate in the pertinent discussions. In particular, community members would benefit from learning more about the benefits of research to their community. Conversely, researchers would be well served when they attend more to local knowledge and priorities within a specific community context. Unlike studies conducted elsewhere, we did not observe significant mistrust of research where “insider-outsider” group dynamics in the relationship between community and researchers was clearly contrasted [53, 54]. This could be partly explained by the fact that locally, there was no apparent legacy regarding mistreatment in research or research injustice [55]. An increasing number of community-based health interventions and research activities in recent years may have also contributed to the perception that such initiatives considered the community as partners working through issues of disparity and inequity in health [56].

It should be noted that while the development of research priorities is important in providing investigative directions, the uptake of final research priorities derived from this exercise may be determined by public funds invested in community health research as well as adequate national policy support for population health. Additionally, given that the vast majority of participants are non-academic stakeholders, the research questions posed in our exercise may not necessarily be scientifically novel; but what is important is that diverse stakeholders were empowered to engage in priority setting, which achieved democratic legitimacy of results.

### Strength and limitations

The main strength of this study is its triangulation of three data sources: leadership feedback, interim survey ranking exercise and semi-structured interviews with individuals from various organisations and professions working in the field of population health. Not only did this triangulation bring greater clarity to the refinement of population health issues, it provided the basis for the development of research priority areas grounded in the experience of stakeholders in the field. In addition, the results of the current research priority exercise represent the perspectives and input from a large group of stakeholders in Singapore. This increases a balanced representation of the stakeholders in the community.

This strength notwithstanding, this study presented several limitations. The categorisation of the priority list based on the CPC feedback was performed by the members of the research team and it is possible that other people would have made different decisions around defining and organising categories. Only one in three symposium participants took part in the ranking exercise resulting in a low response rate. As this exercise was voluntary, we were unable to collect more responses. It is possible therefore that those who did not participate in the ranking exercise may have responded differently. This limitation was counteracted by the subsequent qualitative element of methodologies to ensure that priority setting exercise is not subject to a majority vote. Although we encouraged in-depth interview participants to suggest priority areas other than the list drawn from the earlier phases, there was general hesitance to put forward ideas that had not been considered previously by others. Despite efforts to involve more service users at each phase of the priority setting process, we had a small response rate from this group. Thus it is possible that this may have influenced the themes and research questions generated as priorities. Indeed, the few illustrative quotes from service users may suggest a deference for service providers to determine the research priorities and themes. To distil the verbatim responses into a representative priority, a process of abstraction was required. We might have lost or misunderstood ideas or viewpoints during the process. Formal member validation was not performed, which otherwise could have improved the credibility of findings. However, we presented the findings to a forum of community leaders who affirmed the study findings. Triangulation of the three data sources also ensured that interpretation of the findings was the accurate reflection of participants’ views. Lastly, since our participants were recruited from the four areas of focus (i.e. primary care, mental health, intermediate and long-term care and end of life care), the list of research priorities may not be generalizable to all areas and a wider population.

## Conclusion

This study suggests the importance of involving stakeholders in the identification of strategic priorities for population health research. As beneficiaries of research and development in population health, it cannot be assumed that views of the community will be naturally consonant with those of the researchers and scientific community. It is hoped that the outcomes derived from this exercise will help focus researchers’ efforts through which meaningful gains can be made for population health [57]. Effective dissemination and uptake of these findings are important and should be assessed in terms of the number of population health research projects carried out, developed or funded in the years to come.

## Supporting information

S1 Table COREQ checklist. (DOC)
